# Androgen receptors beyond prostate cancer: an old marker as a new target

**DOI:** 10.18632/oncotarget.2831

**Published:** 2014-11-15

**Authors:** Javier Munoz, Jennifer J. Wheler, Razelle Kurzrock

**Affiliations:** ^1^ Hematology-Oncology, Banner MD Anderson Cancer Center, Gilbert, Arizona; ^2^ Department of Investigational Cancer Therapeutics (Phase I Clinical Trials Program), The University of Texas MD Anderson Cancer Center, Houston, Texas; ^3^ Center for Personalized Cancer Therapy, Division and Hematology and Oncology, Moores Cancer Center, The University of California San Diego, La Jolla, California

**Keywords:** androgen receptor, therapy, molecular abnormalities, phase I clinical trials

## Abstract

Androgen receptors (ARs) play a critical role in the development of prostate cancer. Targeting ARs results in important salutary effects in this malignancy. Despite mounting evidence that ARs also participate in the pathogenesis and/or progression of diverse tumors, exploring the impact of hormonal manipulation of these receptors has not been widely pursued beyond prostate cancer. This review describes patterns of AR expression in a spectrum of cancers, and the potential to exploit this knowledge in the clinical therapeutic setting.

## INTRODUCTION

Treatments targeting androgen receptors (ARs) have demonstrated efficacy in patients with prostate cancer. Despite a wealth of reports indicating that ARs may contribute to the growth and/or progression of numerous other malignancies, their precise role in cancers beyond those of the prostate is poorly understood. The aim of this review is to illuminate current data in the literature regarding the expression and functional impact of ARs in a range of solid tumors (Table 1), and to discuss the therapeutic implications of AR positivity.

**Table 1:** Androgen receptor (AR) expression across malignancies

Malignancy	Subtype	AR positivity % (positive/tested)*	Supplementary References
Adrenocortical carcinoma	Not specified	38% (6/16)	[S49]
Astrocytoma	Grade I/II	28% (5/18)	[S89]
Astrocytoma	Anaplastic	75% (3/4)	[S89]
Basal cell carcinoma	Not specified	65% (20/31) – 78% (25/32)	[S34, S35]
Bladder cancer	Urothelial carcinoma	12.9% (61/472) - 51% (7/139)	[S53, S55]
Breast cancer	Her-2 adenocarcinoma	85.6% (89/104)	[S110]
Breast cancer	BRCA1	32% (43/135)	[S112]
Breast cancer	BRCA2	13% (18/135)	[S112]
Breast cancer	BRCA negative	76% (56/74)	[S112]
Cervical invasive carcinoma	Squamous	23% (3/13)	[S91]
Colon adenocarcinoma	Not specified	38% (5/13)	[S80]
Desmoid tumors	Not specified	52.9% (14/27)	[S85]
Esophageal carcinoma	Not specified	25% (1/4) – 33% (7/21)	[S69]
Gastric carcinoma	Not specified	12.5% (2/16) – 39% (46/117)	[S65, S67]
Glioblastoma multiformis	Not specified	40% (4/10)	[S89]
Head and neck carcinoma	Laryngeal squamous	0% - 10% (1/10)	[S26, S27]
Juvenile nasopharyngeal fibroma	Not specified	38.4% (5/13) - 75% (18/24)	[S22, S23]
Melanoma	Not specified	4% - 40%	[S30]
Meningioma	Not specified	42% (8/19)	[S90]
Non-small cell lung cancer	Not specified	31% (20/64) - 70.5% (12/17)	[S62, S63]
Ovarian cancer	Epithelial	18% (28/154) – 27% (7/26)	[S101, S105]
Ovarian cancer	Steroid cell tumors	64% (9/14)	[S100]
Pancreatic adenocarcinoma	Not specified	0% (0/12)	[S72]
Peritoneal mesothelioma	Not specified	65%	[55]
Prostate cancer	Not specified	95%	[S58]
Testicular germ cell	Seminoma	45%	[S44]
Thymic carcinoma	Not specified	0% (0/12)	[S19]
Thymoma	B3 thymoma	58.8% (10/17)	[S19]
Thyroid carcinoma	Papillary	79% - 80% (4/5)	[S11, 56]
Thyroid carcinoma	Follicular	14% (4/28)	[S11]
Thyroid carcinoma	Medullary	2% - 14% (4/28)	[S11, S12]
Thyroid carcinoma	Anaplastic	1%	[S11]
Rectal adenocarcinoma	Not specified	39% (9/23)	[S80]
Renal cancer	Not specified	14.8% (27/82) – 42% (5/12)	[S45, S46]
Salivary gland	Pleomorphic adenoma	4.8% (2/41) – 100% (14/14)	[S2]
Salivary gland	Salivary duct carcinoma	92% (11/12) – 100% (6/6)	[S1, S2, S4-S6]
Salivary gland	Mucoepidermoid carcinoma	6.6% (2/30) - 20% (2/10)	[S2]
Salivary gland	Adenoid cystic carcinoma	0% (0/8) – 20% (2/10)	[S2]
Sarcoma	Kaposi	0% (0/22)	[S42]
Sarcoma	Osteosarcoma	28.5% (8/28) – 50.8% (33/65)	[S36, S37]
Uterine sarcoma	Leiomyosarcoma	0% (0/28) – 40% (10/25)	[S95, S97]
Uterine sarcoma	Endometrial stromal	45% (9/20)	[S96]
Uterine carcinoma	Endometrial carcinoma	16% (4/25) - 88.6% (39/44)	[S93, S94]

* Numerators and denominators in the third column were given when they were clearly defined in the references.

### Background

#### Androgen synthesis and androgen receptors

The goal of androgen deprivation or suppression therapy is to reduce the levels of male hormones (androgens) [1]. The major androgens capable of stimulating the androgen receptors include testosterone and dihydrotestosterone (DHT) [2]. In normal men, androgens are synthesized in the testes (interstitial Leydig cells) and adrenals. Testosterone is the principal circulating androgen in adult men, with >95% being of testicular origin [3] (under the regulatory control of the hypothalamic / pituitary axis), while the remainder originates from peripheral conversion of weaker precursors of adrenal origin: dehydroepiandrosterone (DHEA), DHEA sulfate, and androstenedione [4]. Adrenal androgens are under adrenocorticotropin hormone (ACTH) control [5]. The adrenals and peripheral conversion/synthesis of androgens do not make a major contribution to androgen effects in the normal man, but may become a significant residual source of AR stimulation in men undergoing androgen deprivation/suppression therapy. Skin, fat, liver, and urogenital systems are important peripheral sites of androgen production [6, 7].

#### Prostate cancer as a model for androgen deprivation therapy

Androgens stimulate AR+ cells to proliferate. Since the vast majority of prostate cancers are AR+, androgen deprivation (suppression) treatment was a rational approach for this malignancy, and has become a cornerstone of treatment for advanced prostate cancer (Table 2) [8]. Androgen deprivation can shrink or stabilize prostate tumors, but is not generally curative. There are protean ways to suppress androgen levels. These include:

- Surgical removal of the testes (castration or orchiectomy).
- Luteinizing hormone-releasing hormone (LHRH) analogs (reversible chemical castration). These drugs halt testosterone production by the testicles.
  Examples of these agents include leuprolide, goserelin, triptorelin, and histrelin [9]. When LHRH analogs are initiated, testosterone levels rise before falling, a phenomenon known as a “clinical flare” [10]. The flare can cause bone pain or spinal cord compression if tumors are present in these areas, as the tumors may have a short-lived growth spurt. The flare can be prevented by giving anti-androgens for a few weeks when LHRN analogs are started.
- Luteinizing hormone-releasing hormone (LHRH) antagonists. LHRH antagonists such as degarelix work like LHRH agonists, but they reduce testosterone levels more rapidly and do not induce a tumor flare like the LHRH agonists do [11].
- Anti-androgens. Anti-androgens impede the body's ability to use androgens, usually by blocking the androgen receptor. Even after orchiectomy or during treatment with LHRH analogs, the adrenal glands still synthesize small amounts of androgens. Drugs of this type include flutamide, bicalutamide, and nilutamide [12].
- Other androgen-suppressing drugs. Estrogens were once the main alternative to orchiectomy for men with advanced prostate cancer. Because of their toxic effects [13] (including blood clots and breast enlargement), oral estrogens have been largely replaced by LHRH analogs and anti-androgens [14]. It has been suggested that parenteral estrogen may avoid long term toxicity [15]. Ketoconazole, first used for treating fungal infections, blocks production of androgens. It offers a quick way to lower testosterone levels, and it is used in countries where abiraterone has yet to be approved [16]. Abiraterone inhibits 17 α-hydroxylase/C17,20 lyase (CYP17A1), an enzyme that is expressed in testicular, adrenal, and prostatic tumor tissues. CYP17 catalyzes key reactions in the testosterone synthesis pathway; inhibition of CYP17 [17] activity by abiraterone thus interferes with the processes in the testes and the adrenals leading to testosterone production.

Androgen deprivation has important side effects [18, 19], especially in men: reduced or absent libido, impotence; hot flashes, gynecomastia, osteoporosis and fractures, attenuated mental acuity, loss of muscle mass, depression, and fatigue

**Table 2:** Examples of Anti-Androgens Approved by the Food and Drug Administration (FDA) agents to treat prostate cancer*

Drug name	Mechanism of action	Indication Examples
Leuprolide acetate	GnRH** agonists	Palliative treatment of advanced prostatic cancer.
Goserelin acetate	GnRH agonists	Palliative treatment of advanced prostatic cancer.
Triptorelin pamoate	GnRH agonists	Palliative treatment of advanced prostatic cancer.
Histrelin acetate	GnRH agonists	Palliative treatment of advanced prostatic cancer.
Degarelix acetate	GnRH antagonists	Palliative treatment of advanced prostatic cancer.
Bicalutamide	Binds to androgen receptor	Metastatic prostate cancer
Flutamide	Competes for AR the and dihydrotestosterone for the androgen receptor	“””
Nilutamide	Blocks AR	Advanced prostate cancer
Abiraterone acetate	CYP17A1 inhibitor	Metastatic castration-resistant prostate cancer who have received prior docetaxel.
Enzalutamide	Androgen receptor inhibitor	Metastatic castration-resistant prostate cancer who have received prior docetaxel.
Radium Ra 223 dichloride	Alpha-particle emitting	Symptomatic bone metastases and no known visceral metastatic disease.

* Data from www.fda.gov (Accessed August 11, 2014).

** AR = androgen receptor; GnRH: gonadotropin releasing hormone.

### THE ROLE OF ANDROGENS IN DIVERSE TUMORS

Although the historic emphasis has been on prosecuting androgen receptors in prostate cancer, it turns out that androgens have a vital role in numerous other cancers as well (Supplemental references 1-119). It is conceivable that interrogation of androgen receptor positivity and treatment with androgen antagonistic agents could therefore be an effective strategy in these cancers.

#### I. HEAD AND NECK CANCERS

##### Salivary gland tumors

Remarkable similarities between breast cancer and salivary gland tumors have been documented [S1-S3]. Not only is HER2/*neu* expression frequent in these tumors, but estrogen receptors (ER) and progesterone receptors (PR) are expressed in 33.3% (13/69) and 86.9% (60/69), respectively, in the pleomorphic adenoma subtype [S3]. AR positivity has also been documented in salivary duct carcinomas [S1, S4-S6]. Nasser et al. [S2] reported a series of 78 formalin-fixed, paraffin-embedded salivary gland tumors with strong positivity for AR in 100% (14/14) of carcinoma ex-pleomorphic adenomas and 100% (6/6) of salivary duct carcinomas. Fan et al. [S5] reported AR positivity rates as high as 92% (11/12) in salivary duct carcinomas. AR reactivity was also seen in 20% (2/10) of acinic cell carcinomas, 20% (2/10) of mucoepidermoid carcinomas, and 20% (2/10) of adenoid cystic carcinomas [S2]. In the same study all 26 benign salivary gland tumors were negative for expression of AR [S2].

In regard to treatment, Locati et al. [S7] reported a complete remission with androgen deprivation therapy in a patient with recurrent AR-expressing adenocarcinoma of the parotid gland. Kuroda et al. [S8] reported a partial response after one course of anti-androgen therapy and palliative chemotherapy with paclitaxel in a patient with advanced salivary duct carcinoma.

##### Thyroid carcinoma

Thyroid cancer is the most frequently occurring endocrine-related malignancy [S9, S10]. The most common form of thyroid cancer is papillary (~79%), followed by follicular (~14%), medullary (~2%), anaplastic (~1%) and other histological subtypes (~4%) [S11]. There are significant rates of recurrence with current therapeutic approaches such as surgery or radioactive iodine therapy [S9]. Because both benign and malignant thyroid lesions are more common in women than in men, it has been suggested that sex hormones play a role. Certainly somatic mutations in *BRAF*, *RAS* and *RET*, all described in thyroid cancer, are not known to account for the gender disparity [S10].

Bléchet et al. [S12] showed AR positivity by immunohistochemistry (IHC) in 14% (4/28) of medullary thyroid carcinomas. Testosterone increased dose-dependent proliferation of AR+ human papillary thyroid cancer cell lines; whereas flutamide, inhibited testosterone-induced cell proliferation [S13]. Wiseman et al. [S14] examined differentiated thyroid cancer and found AR+ in 50.5% (50/99) compared to 2% (2/100) of benign lesions. Prinz et al. [S15] showed AR+ in 17 of 31 cases of thyroid cancer, including 80% (4/5) of papillary carcinomas and 80% (4/5) of follicular adenomas. Despite high AR expression in thyroid cancer, there are only anecdotal reports of therapeutic hormonal manipulation [S16].

##### Thymoma and thymic carcinoma

Ishibashi et al. [S17] examined patients with thymoma and found AR+ nuclear immunoreactivity by IHC in 15% of patients (20/132). Mimae et al. [S18] studied thymic epithelial tumors (103 thymomas and 37 thymic carcinomas) and found that 23.6% expressed AR. The World Health Organization (WHO) classified epithelial thymic tumors into six types including A, AB, B1, B2, B3, and thymic carcinoma. Because the histological differential diagnosis between type B3 thymoma and thymic carcinoma can be challenging, Khoury et al. [S19] proposed that AR might be useful for pinpointing the diagnosis, particularly because cytoplasmic AR was found in 58.8% (10/17) of type B3 thymomas versus 0% (0/12) of thymic carcinomas [S19]. Preclinical data suggest that blocking AR with 5-alpha-dihydrotestosterone can initiate apoptosis in thymoma cells [S20].

##### Juvenile nasopharyngeal angiofibroma

Several publications have documented that these benign hormone-dependent tumors, particularly the ones prone to affecting adolescent males, express AR [S21, S22]. IHC results showed AR+ in 75% (18/24) of nasopharyngeal angiofibroma cases [S23]. Montag et al. [S24] showed that AR is expressed in 38.4% (5/13) of patients. These results translated to the clinic where 4 of 5 patients with juvenile nasopharyngeal angiofibroma demonstrated an average shrinkage of 44% following flutamide [S25].

##### Head and neck carcinomas

Bianchini et al. [S26] studied tissue from 15 patients with laryngeal carcinoma but found no expression of AR (0%) [S26]. Virolainen et al. [S27] found 10% (1/10) AR positivity in cell lines derived from laryngeal squamous cell carcinoma and 0% (0/9) AR positivity in non-laryngeal head and neck squamous tumors. Mattox et al. [S28] evaluated flutamide in patients with laryngeal carcinoma and, surprisingly, short-lived partial responses were seen in 3 of 9 patients. AR levels did not correlate with response [S28].

#### II. CUTANEOUS MALIGNANCIES

##### Melanoma

AR reactivity was present in 40% of melanomas; however, it was also observed in 41% of normal skin [S29]. In contrast, a different report (N=142 patients with malignant melanoma) discerned AR positivity in only 4% of cases [S30]. Morvillo et al. [S31] showed ARs in human melanoma cell lines, with tumor growth after exposure to dihydrotestosterone versus significant inhibition of proliferation after tamoxifen or flutamide [S32]. Ketoconazole, an androgen synthesis inhibitor, was shown to abrogate the metastatic potential of melanoma cell lines in mice [S33].

##### Basal cell carcinoma

Katona et al. [S34] and Izikson et al. [S35] reported AR expression in 65% (20/31) and 78% (25/32), respectively, of basal cell carcinoma cases; they suggested that the expression was high enough to help distinguish basal cell carcinoma from other skin conditions with low or negative AR expression, such as benign trichoblastic tumors and desmoplastic trichoepithelioma.

#### III. SARCOMA

Limited studies of AR in sarcomas have been performed.

##### Osteosarcoma

AR+ has been demonstrated in 28.5% (8/28) to 50.8% (33/65) of osteosarcomas [S36,S37]. Proliferation of osteosarcoma cell lines *in vitro* was stimulated by estradiol, progesterone, and 5 alpha-dihydrotestosterone; whereas growth was abrogated by AR antagonists as fulvestrant, mifepristone, and hydroxiflutamide. Interestingly, higher levels of AR correlated with a higher degree of differentiation. In preclinical models, the adrenal androgen synthesis inhibitor ketoconazole prompted apoptosis in human osteosarcoma cells [S38].

##### Other malignant sarcomas

We were unable to find AR expression data regarding gastrointestinal stromal tumors in the literature [S39, S40]. In 1980, Walker et al. [S41] reported steroid receptors in malignant skeletal malignancies, including four cases of osteosarcoma, two chondrosarcomas, one malignant giant cell tumor, and one Ewing's sarcoma. Cytoplasmic AR and PR were expressed in three of seven cases (43%) [S41]. Ziegler et al. [S42] found 0% (0/22) AR expression in tissue from 22 patients with Kaposi's sarcoma.

#### IV. GENITOURINARY MALIGNANCIES

##### Testicular germ cell tumors

There was no AR staining in embryonal carcinoma, mature teratoma, seminoma, or mixed germ cell tumors, although trace AR expression was found in 3 out of 5 cases of endodermal sinus tumors [S43]. In a small study of seminomas, AR expression was detected in 45% of cases (N = 18) [S44].

##### Renal cell carcinoma

AR expression has been demonstrated in 14.2% (3/21), 14.8% (27/182) and 42% (5/12) of renal cell carcinomas [S45-S47]. AR positivity was associated with a significantly better progression-free survival (PFS) compared to AR negativity in renal cancer [S45]. Brown et al. [S46] demonstrated AR immunoreactivity in 5 of 12 of patients with primary and in 1 of 5 of patients with metastatic clear cell renal cell carcinoma. Based on a small number of patients, Nakano et al. [S15] hypothesized that hormonal manipulation exerted no antineoplastic effect *vis-a-vis* tumor shrinkage, but did prolong survival in the subgroup with hormonal receptor positivity. A phase II trial of flutamide, a nonsteroidal antiandrogen, in 25 patients with advanced renal cell cancer, demonstrated one partial response for more than nine months and two patients maintained stable disease for six and 15 months, respectively [S15]. AR expression was seen in 25% (3/12) of biopsied patients.

##### Adrenocortical carcinomas

Because chemotherapy is effective in only a minority of patients with adrenocortical carcinomas, steroidogenesis inhibitors such as ketoconazole, mitotane, etomidate and metyrapone, have been used as single agents or combined with cytotoxic chemotherapy [S48]. Barzon et al. [S49] found that 38% (6/16) of patients with adrenocortical carcinoma were AR+ by IHC analysis. Rossi et al. [S50]] demonstrated AR RNA in human adrenocortical cancer cell lines. Ketoconazole reduces adrenal steroid biosynthesis by inhibiting cytochrome P450-dependent adrenal enzymes and is effective in reducing hormonal levels in virilizing adrenocortical carcinomas. However, generally no tumor shrinkage has been seen [S51, S52].

##### Bladder cancer

AR positivity has been found in 13% to 51% of bladder tumors [S53-S55] but not in normal bladder tissue [S55]. Loss of AR correlated with higher grade tumors [S55]; on the other hand, AR positivity was more frequent (71%) in metastatic disease than in primary tumors [S54]. There was no significant difference in the rate of AR positivity between men and women, despite the fact that bladder cancer is more common in men [S53]. Androgen blockage by flutamide *in vitro* as well as *in vivo* in mouse xenograft models with human AR-expressing bladder cancer cell lines attenuated proliferation and tumor growth [S56]. Hameed et al. [S57] assessed the combination of ketoconazole and all-trans retinoic acid in 16 patients with superficial bladder cancer, and demonstrated tolerable toxicity and reduced recurrence rate, albeit in the context of a small uncontrolled study.

##### Prostate cancer

The discussion of prostate cancer herein is limited as AR and prostate cancer has been reviewed extensively elsewhere. Briefly, prostate cancer is a prime example of the potential for therapeutically manipulating hormonal levels (Table 2). This is feasible because strong nuclear AR reactivity is the norm in more than 95% of the samples studied [S58]. There is a high correlation between AR expression and response to hormonal treatment, and anti-androgens are therefore the mainstay of therapy for patients with prostate cancer [S59, S60]. Frequent AR positivity is found in more highly differentiated versus poorly differentiated tumors; further, AR was rarely seen in prostate tumors of patients who had received long-standing hormonal therapy [S61].

#### V. LUNG MALIGNANCIES

##### Non-small cell lung cancer

Kaiser et al. [S62] found AR expression in 70.5% (12/17) of non-small cell lung cancer cases. Rades et al. [S63] retrospectively found 31% (20/64) AR positivity in patients who received radiation for stage II/III non-small cell lung cancer.

##### Small cell lung cancer

Kaiser et al. [S62] mentioned that hormonal receptor expression was almost non-existent in the small cell lung cancer cell lines. Maasberg et al. [S64], however, demonstrated growth stimulation by testosterone and growth inhibition by flutamide in selected AR+ small cell lung cancer cell lines.

#### VI. GASTROINTESTINAL MALIGNANCIES

##### Gastric carcinomas

AR positivity has been found in 12.5% (2/16) to 39% (46/117) of gastric cancers [S65-S67]. AR positivity may correlate with poorer survival [S66].

##### Esophageal carcinomas

Yamashita et al. [S68] found AR positivity in two cases of human esophageal cancer xenografts implanted into nude mice; tumor progression was fueled by testosterone and inhibited by castration. Tihan et al. [S69] documented AR reactivity in 45% (5/11) of adenocarcinomas and 21.4% (3/14) of squamous cell carcinomas of the esophagus, respectively. Esophageal cancer seems to be more frequent in men than in women. However, no difference in the rate of positivity between genders was seen, though the numbers of patients were small, precluding definite conclusions. Interestingly, survival was similar regardless of AR status [S69]. Of possible interest, the Survival Epidemiology and End Results (SEER) registries database showed that patients with prostate cancer are less likely to develop esophageal adenocarcinoma [S70]. The relationship between these facts and treatment with androgen deprivation remains unexplored.

##### Pancreatic adenocarcinomas

Tumor progression in a nude mice xenograft model was stimulated by testosterone and inhibited by the anti-androgen cyproterone acetate [S71]. However, Targarona et al. [S72] found no AR expression in 12 biopsies of exocrine pancreatic neoplasia. A phase II study of the anti-androgen flutamide in an unselected population of 14 patients with advanced pancreatic adenocarcinoma yielded no objective responses [S73]. Subsequently, a randomized trial showed encouraging prolongation of median survival to 8 months in the flutamide group compared to 4 months in the placebo group [S74]. A counterpoint, however, is the fact that patients with advanced pancreatic carcinoma have lower than expected testosterone secondary to malnutrition and consumption [S75]. In a preclinical model Konduri et al. [S76] did not show any significant improvement from combining flutamide with gemcitabine or bevacizumab. Since estrogen receptors may also be expressed in pancreatic cancer; the relationship between androgen and estrogen, when designing clinical trials testing hormonal blockade, will be particularly important in malignancies that express both receptors [S77, S78].

##### Colorectal adenocarcinomas

Tutton et al. [S79] confirmed that colon tumor cell proliferation was enhanced by testosterone and cell growth was attenuated by flutamide. Stebbing et al. [S80] found cytosolic AR in 39% (9/23) of rectal and 38% (5/13) of cecal adenocarcinomas. Several authors have described AR positivity in almost all samples of both colon tumors and normal mucose [S81, S82], while others have shown smaller, but still significant, percentage of AR positivity (32% colon tumors; 67% non-tumor tissue) [S83]. Further, ketoconazole has antiproliferative effects in human colon cancer cell lines [S84]. In preclinical rat models, cell proliferation in colon tumors was triggered by testosterone and inhibited by flutamide [S79]. However, the fact that normal colorectal mucosa expresses AR might blunt the usefulness of AR inhibitors in colorectal cancer.

#### VII. CONNECTIVE TISSUE TUMORS

##### Desmoid tumors

Ishizuka et al. [S85] documented AR expression in 52.9% (14/27) of patients with desmoid tumors. Goserelin, a gonadotropin releasing hormone agonist, resulted in stable disease in two patients with desmoid tumors for 4 and 16 months, respectively [S86]. Bauernhofer et al. [S87] reported a case of inoperable intra-abdominal desmoid tumor that decreased in size after treatment with goserelin acetate and low-dose tamoxifen for 17 months.

#### VIII. CENTRAL NERVOUS SYSTEM MALIGNANCIES

##### Glioblastoma multiforme and other intracranial tumors

In preclinical studies, testosterone stimulated proliferation of glioblastoma multiforme cell lines [S88]. Chung et al. [S89] documented AR positivity in 40% (4/10) of glioblastoma multiforme cases, 28% (5/18) of grade I/II astrocytomas and 75% (3/4) of anaplastic astrocytoma. Lee et al. [S90] found AR+ in 42% (8/19) of patients with meningioma.

#### VIII. GYNECOLOGIC MALIGNANCIES

##### Cervical cancer

One study demonstrated the expression of AR by immunohistochemistry in 100% (30/30) of normal epithelium, 100% (30/30) of low-grade cervical intraepithelial neoplasia, and 63% (19/23) of high-grade cervical intraepithelial neoplasia, as well as in 23% (3/13) of invasive squamous cell carcinoma. The authors suggested the loss of AR expression is a common occurrence in malignant cervical transformation [S91]. Even so, the fact that significant subsets of gynecologic cancers express AR may have clinical relevance.

##### Uterine cancer

Yang et al. [S92] found that AR positivity was the most robust variable associated with the risk of endometrial cancer. Ito et al. [S93] found AR reactivity in 88.6% (39/44) of endometrial carcinomas. Brys et al. [S94] found AR expression in 8% (1/12) of normal endometrium compared to 16% (4/25) of endometrial cancers. The reasons for this variability may relate to lack of standardized methodology.

##### Uterine sarcoma

A review of 65 cases of uterine sarcoma revealed 0% (0/65) AR+ [S95]. In contrast, Moinfar et al. [S96] found AR reactivity in 45% (9/20) of malignant endometrial stromal neoplasms. As benign leiomyomas are responsive to hormonal manipulation; Leitao et al. [S97] showed AR expression in 32% (6/19) of benign uterine leiomyoma and in 40% (10/25) of uterine leiomyosarcoma. AR positivity was predictive of a lower risk of recurrence although, after controlling for stage, AR was not significantly associated with overall survival improvement [S97].

##### Ovarian cancer

Testosterone binding sites have been found in 43% of normal ovaries, 40% of benign ovarian tumors, and 60.5% of ovarian adenocarcinomas [S98]. Elattar et al. [S99] noted that AR-expressing epithelial cells in ascitic fluid from patients with ovarian cancer were stimulated by dihydrotestosterone and inhibited by bicalutamide. AR expression was significantly reduced in paired samples after chemotherapy which might explain the lack of response to hormonal blockade in heavily pre-treated patients [S99]. Jones et al. [S100] studied steroid cell ovarian tumors, finding AR immunoreactivity in 64% (9/14) of samples. High AR expression in 18% (28/154) of epithelial ovarian cancer was reported [S101]. Patients with AR-positive serous tumors had improved disease-specific survival [S101]. Additionally, reports of transsexuals who developed ovarian cancer in the setting of androgen supplementation suggest a carcinogenic role for AR [S102]. In regard to treatment, a phase II trial of goserelin and bicalutamide in 35 patients with epithelial ovarian cancer did not prolong PFS [S103, S104]. Because chemotherapy reduces AR expression, anti-androgen therapy may be more effective early in the disease [S104]. One study demonstrated dissimilar AR reactivity within a single ovarian neoplasm, attesting to tumor heterogeneity [S105]. In a phase II trial, 32 patients with advanced ovarian neoplasia received a minimum of two months of flutamide resulting in one complete response for 73 weeks, one partial response for 44 weeks, and partial responses in 28% (9/32) for a median of 24 weeks [S106]. A case report documented a patient with Leydig cell ovarian tumor, an androgen-producing neoplasm, which responded to the GnRH-analogue triptoreline [S107]. Because androgen receptors are frequently expressed in epithelial ovarian cancer, investigation of newer anti-androgens in this disease may be worthwhile [S108].

#### VIII. BREAST CANCER

Peters et al. studied invasive breast ductal adenocarcinomas, finding 56% AR positivity by IHC, and expression was inversely associated with 10-year survival [S109]. In HER2/−positive breast malignancies, AR expression was found in 85.6% (89/104). AR-negative/estrogen receptor-negative was the most aggressive phenotype and correlated with high-grade tumors [S110]. Similarly, Hu et al. [S111] documented AR positivity in 78.7% of 1,467 patients with breast cancer. Pristauz et al. [S112] studied the relationship between AR and BRCA mutations in a population of patients constituted by 32% (43/135) BRCA1 and 13% (18/135) BRCA2. Interestingly, AR reactivity was seen in 30% (13/43) of BRCA1 and in 78% (14/18) in BRCA2, suggesting that many ER−/PR− BRCA-mutant tumors are actually AR positive [S112]. A higher number of breast carcinomas were found to express AR (77%) compared to ER (61%) and PR (60%) in 980 cases, with more than 50% of triple negative tumors expressing AR [S113]. The fact that AR+ was lost by the time of autopsy (5/11) implies loss of AR expression in end-stage metastases [S114]. From a molecular standpoint, Collins et al. [S115] studied 2,171 invasive breast malignancies with tissue microarrays, dividing them into luminal-A (64%), luminal-B (15%), basal-like (11%), and HER2 (6%). AR expression was seen in 77% of invasive breast malignancies divided into luminal-A (91%), luminal-B (68%), basal-like (32%), and HER2 (59%). Interestingly, AR expression was seen in 86% of 246 cases of ductal carcinoma *in situ* [S115]. Breast cancer cell lines may be at least AR-dependent [S116, S117]. Other preclinical studies in human breast cancer cell lines showed that AR overexpression induced tamoxifen resistance which was reversed by the AR antagonist bicalutamide [S118]. In regard to treatment, Gucalp et al. [S119] report on a multicenter phase II trial bicalutamide for patients with AR+/ER−/PR− breast cancer showed a clinical benefit rate of 19%.

## DISCUSSION

### Therapeutic implications of AR across malignancies

**Figure 1:**
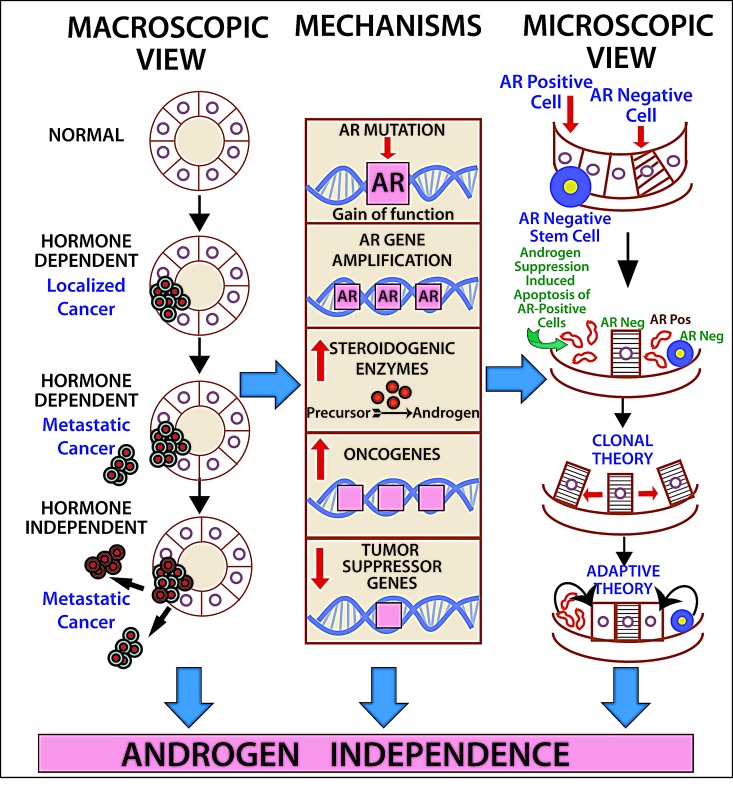
The road to (androgen) independence AR-negative prostate adult stem cells generate AR-negative transit amplifying cells, AR-negative intermediate cells and AR-positive luminal secretory cells. Once therapeutic circulating androgen blockade ensues, AR-positive luminal cells die, giving origin to the adaptive (selected AR-positive luminal cells will continue to thrive despite lack of androgen exposure) and clonal theory of resistance (pre-existent AR-negative cells develop into a malignant clone). Castration-resistant prostate cancer might develop via ligand-dependent (tissue steroidogenesis, AR mutations, AR amplification) and ligand-independent pathways (heightened AR nuclear translocation, AR cross-talk with additional pathways, disturbing the balance between co-activators and co-repressors).

**Figure 2:**
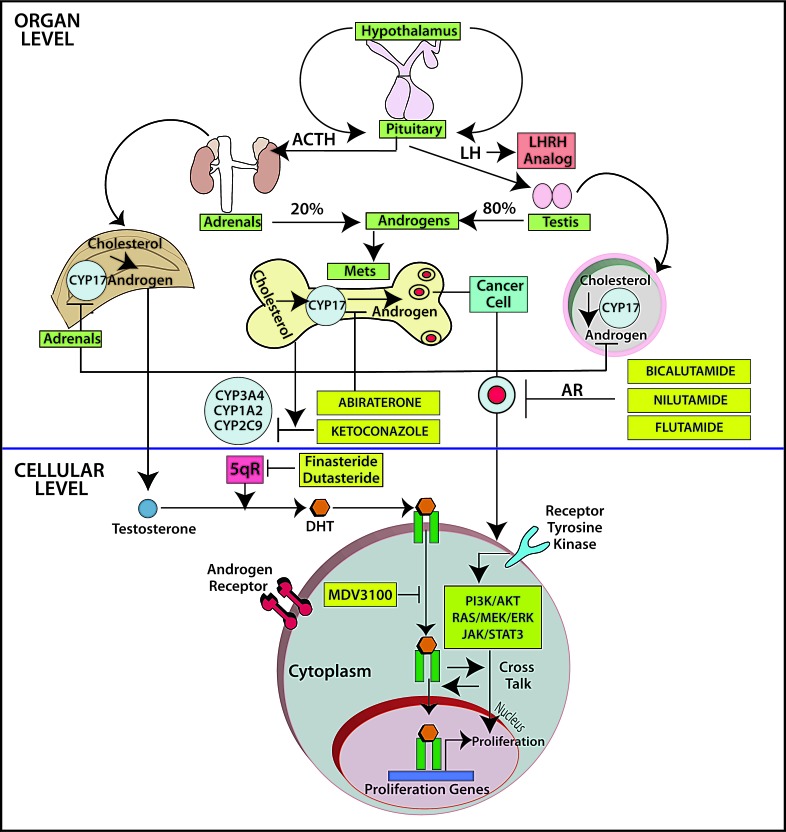
The fate of testosterone in prostatic tissues Testosterone circulates in the blood and is bound to albumin, whereas free testosterone is introduced into prostate cells and is subsequently converted to DHT by 5-alpha-reductase. Binding of DHT to the AR induces dissociation from HSPs and receptor phosphorylation. The AR dimerizes and can bind to androgen-response elements in the promoter regions of target genes, leading to growth, survival and production of PSA. Enzalutamide, formerly called MDV3100, exerts its mechanism of action during several steps in the AR signaling pathway including inhibition of AR binding to androgens, inhibition of nuclear translocation of AR, inhibition of AR association to DNA, and AR amplification. As some of those aberrations may occur late in the disease, it is unknown at this point if enzalutamide will have a role upfront in the management of prostate cancer. Abbreviations: AR, androgen receptor; DHT, dihydrotestosterone; GTA, general transcription activation; HSP, heat-shock protein; SHBG, sex-hormone-binding globulin; AKT, akt serine/threonine kinase; DHEA, dihydroepiandrosterone; ERK, extracellular signal-regulated kinase; P, phosphorylated residues; PI3K, phosphoinositide 3-kinase; PTEN, phoshatase and tensin homolog.

**Figure 3:**
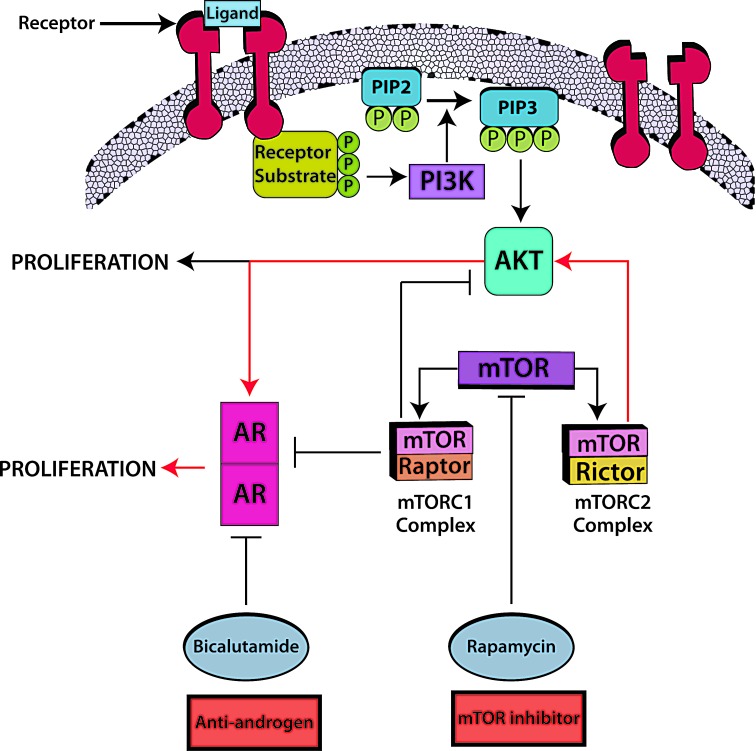
Androgen blockade plus mTOR inhibitors: A prime candidate for combination hormonal treatment The mTORC2/AKT/AR pathway (red arrows) leads to tumor cell proliferation. Rapamycin combined with bicalutamide has an apoptosis-inducing effect in prostate cancer. Rapamycin inhibits both mTOR complexes, mTORC1 with raptor and mTORC2 with rictor; nevertheless abrogation of mTORC2, a kinase for AKT phosphorylation, further inhibits the AR transcription cascade in an AKT-dependent manner. As a counterpoint, abrogation of mTORC1 produces AKT/AR-independent apoptosis, though it continues to stimulate the AR transcriptional cascade and AKT phosphorylation. According to the suggested cross-talk, it would take a combination of androgen blockade plus mTOR inhibitors to fully abrogate the mTORC2/AKT/AR pathway. Barnett et al. [44] found that, out of 47 tumors evaluable by immunohistochemistry, 36% had PTEN loss which was associated with an increased relapse in high risk prostate cancer treated with chemotherapy followed by surgery. PTEN loss activates the AKT/mTOR pathway [45] thus supporting the use of mTOR inhibitors in this condition. Abbreviations: AR, androgen receptor; AKT, aKT serine/threonine kinase.

Lessons from prostate cancer: During the last several decades, androgen deprivation (Table 2, Figure 1) has been standard of care for metastatic prostate cancer; however, most patients eventually develop disease progression despite pharmacologic suppression of testosterone levels [20-22], denoted as castration-resistant prostate cancer (Figure 2) perhaps mediated by androgen independence (Figure 1) [23, 24]. The goals of hormonal deprivation therapy for prostatic cancer are to decrease circulating plasma testosterone to castration levels and to block residual androgen at the cellular level (Figure 3). While orchiectomy is very effective at achieving some of these goals, it does not halt conversion of residual adrenal androgens to dihydrotestosterone (DHT). Certainly, the common examples of potent androgens include testosterone and DHT, which can directly stimulate androgen receptors. Subsequent therapies were added to the hormonal armamentarium including, but not limited to, bicalutamide, a competitive non-steroidal androgen receptor antagonist; flutamide, a non-steroidal anti-androgen; and luteinizing hormone-releasing hormone (LHRH) or gonadotropin-releasing hormone (Gn-RH) [25]. Leydig cells, which are located in the testes, are dependent on LH to produce and secrete testosterone. Gn-RH is a hypothalamic decapeptide that governs the synthesis of pituitary LH; thus synthetic Gn-RH analogues were initially developed to treat infertility in cases of endogenous Gn-RH deficiency. Long-term administration of supra-physiologic Gn-RH produce the paradoxical effect of pituitary overstimulation including pituitary desensitization to Gn-RH, breakdown of physiological feedback systems, down-regulation of Gn-RH receptors, depletion of releasable LH content, and reduction of testosterone secretion to castrate levels. It is not, therefore, surprising that high-dose Gn-RH analogues became a major therapeutic option in prostate cancer, as they bypass the need for orchiectomy [26]. Exogenous estrogens indirectly affect the prostate by disturbing the hypothalamus-pituitary-testes axis as estrogens inhibit the release of Gn-RH from the hypothalamus [27]. Recently, the realization that non-gonadal sources of androgens, such as the adrenal glands and intra-tumoral production, might be critical in the progression of prostate cancer led to the development of abiraterone acetate, an oral specific inhibitor of CYP17A1, which is a rate-limiting enzyme in androgen (and estrogen) synthesis that, when targeted, specifically suppresses adrenal androgens [28]. A novel alternative is the use of intermittent androgen suppression which holds the promise of decreasing toxicity while maintaining efficacy, although the role of intermittent therapy in the metastatic setting remains a challenge [29]. Hussain et al. [30] has studied intermittent versus continuous androgen deprivation in hormone sensitive metastatic prostate cancer patients in a phase III trial and their published final results are eagerly awaited. In the non-metastatic setting, Crook et al. [31] confirmed that intermittent androgen deprivation showed non-inferior overall survival when compared to continuous therapy after prostate irradiation.

Finally, there are novel therapeutic agents including the once-daily androgen receptor signaling inhibitor enzalutamide, previously called MDV3100 [32, 33] (Figure 3), which significantly prolonged overall survival in a randomized phase III trial that involved 1,199 men with castration-resistant prostate cancer [34]. Such results triggered the Food and Drug Administration to approve enzalutamide in August of 2012 for the treatment of patients with metastatic castration-resistant prostate cancer who have previously received docetaxel. The recent wave of novel anti-androgen agents, as abiraterone and enzalutamide, serves as proof-of-principle that AR signaling continues even in the so-called castration-resistant prostate cancer population [35].

### Tolerance of Anti-Androgens

Hormonal therapy is not devoid of side effects, and depending on the agent used, toxicity can include increased risk of fractures, increased lipoproteins, decreased insulin sensitivity, increased cardiovascular disease, low libido and emasculation [36]; and such toxicity must be kept in mind during the design of future clinical trials.

### Overcoming resistance to AR targeted therapy: Relevant molecular pathways

The mTOR (mammalian target of rapamycin) pathway has been hailed as a possible target for suppressing prostate malignancies (Figure 3), as the PI3K/AKT components of the pathway are upregulated in hormone-refractory prostate neoplasia, and because the combination of rapamycin and bicalutamide produces apoptosis in prostate cell lines [37-39]. Indeed, this pathway is deregulated in up to 65% of prostate cancers, most commonly due to PTEN loss, and less commonly related to PIK3CA amplification or mutation [40]. Wang et al. described the regulation of androgen receptor transcriptional activity by rapamycin in prostate cancer cell proliferation and survival [38]. Furthermore, there seems to be cross-talk between AR and the epidermal growth factor receptor (EGFR) (Figure 3), which in turn activates the expression of mTOR [41]. Gonzalez-Angulo et al. [42] found significantly higher AR levels in breast cancer patients with kinase domain PIK3CA mutations versus wild-type PIK3CA. Wang et al. [43] hypothesized that AR halts PTEN transcription in the prostate in contrast to the breast, in which AR promotes PTEN transcription. Barnett et al. [44] found that, out of 47 tumors evaluable by IHC, 36% had PTEN loss which was associated with an increased relapse in high risk prostate cancer treated with chemotherapy followed by surgery. PTEN loss activates the PI3K/AKT/mTOR pathway [45] thus supporting the use of mTOR inhibitors in this condition.

While both EGFR and AR directly stimulate cell growth, AR apparently indirectly exerts stimulation of EGFR synthesis by paracrine or autocrine mechanisms [46]. In addition to AR-EGFR crosstalk, Src kinase is implicated in EGFR phosphorylation [47]. Meanwhile, interaction between AR and the MAPK pathway has been demonstrated with HER2-AR-ERK feedback loops in breast cancer [48]. Naderi et al. has documented synergy between flutamide and the HER2 inhibitor AG825; and subsequently, synergy between flutamide and the MEK inhibitor CI-1040 [49, 50]. In a preclinical model, DHT enhanced IL-6 and IL-8 expression and flutamide abrogated IL-6 and IL-8 expression [51]. Darshan et al. suggested that taxanes, microtubule stabilization agents, are active in castrate-resistant prostate cancer and act by inhibiting AR trafficking and the downstream cascade of transcriptional events, including AR target genes such as prostate-specific antigen [52]. Testing this concept, Kuroda et al. [S8] treated a patient with metastatic AR-positive salivary duct carcinoma with combined anti-androgen treatment and paclitaxel, achieving a partial response.

Other mechanisms promoting resistance may include genetic and epigenetic adaptation, clonal selection, and evolution of the tumor microenvironment in prostate cancer. The synthesis of constitutively active AR variants lacking the canonical ligand-binding domain may also promote resistance [53].

#### FUTURE DIRECTIONS

AR is ubiquitously expressed across malignancies. Drawing definitive conclusions about rates of expression is however challenging, mainly due to the lack of standardized AR measurement methods and the relatively small number of patients tested for AR expression. Importantly, there is a paucity of studies of androgen manipulation in AR+ tumors other than prostate cancer. Importantly, some tumors such as salivary gland ductal tumors have AR positivity rates approaching 100%. Anecdotal reports and small studies in a variety of malignancies suggest that AR-positive tumors may respond to hormonal manipulation. Of interest, breast and gynecologic tumors also have high rates of AR positivity (Table 1). The latter may have special clinical relevance [54], especially since aromatase inhibitors, used to suppress estrogen levels in patients with ER+ breast tumors, can raise testosterone levels. As an example, anastrozole increases testosterone levels by decreasing serum estradiol levels [55]. Finally, PIK3CA/AKT/mTOR signaling is ubiquitously deregulated in cancer, and it is apparent that there is significant crosstalk between this pathway and that related to androgens (Figure 3). Further exploration of androgen modulation in patients with diverse cancers merits investigation.

## SUPPLEMENTAL REFERENCES


