# Supplementary material for: Androgen receptors beyond prostate cancer: an old marker as a new target
Source: Oncotarget. 2014 Nov 15;6(2):592–603. doi: 10.18632/oncotarget.2831 (PMC4359241; doi:10.18632/oncotarget.2831)
Supplement: Supplementary file 1 [file oncotarget-06-592-s001.pdf]

Androgen receptors beyond prostate cancer: an old marker as a new target

SUPPLEMENTAL REFERENCES

1. Ito FA, Ito K, Coletta RD, *et al.* Immunohistochemical study of androgen, estrogen and progesterone receptors in salivary gland tumors. Braz Oral Res 2009;23(4):393-8.
2. Nasser SM, Faquin WC, Dayal Y. Expression of androgen, estrogen, and progesterone receptors in salivary gland tumors. Frequent expression of androgen receptor in a subset of malignant salivary gland tumors. Am J Clin Pathol 2003;119(6):801-6.
3. Glas AS, Hollema H, Nap RE, *et al.* Expression of estrogen receptor, progesterone receptor, and insulin-like growth factor receptor-1 and of MIB-1 in patients with recurrent pleomorphic adenoma of the parotid gland. Cancer 2002;94(8):2211-6.
4. Moriki T, Ueta S, Takahashi T, *et al.* Salivary duct carcinoma: cytologic characteristics and application of androgen receptor immunostaining for diagnosis. Cancer 2001;93(5):344-50.
5. Fan CY, Melhem MF, Hosal AS, *et al.* Expression of androgen receptor, epidermal growth factor receptor, and transforming growth factor alpha in salivary duct carcinoma. Arch Otolaryngol Head Neck Surg 2001;127(9):1075-9.
6. Jaspers HC, Verbist BM, Schoffelen R, *et al.* Androgen receptor-positive salivary duct carcinoma: a disease entity with promising new treatment options. J Clin Oncol 2011;29(16):e473-6.
7. Locati LD, Quattrone P, Bossi P, *et al.* A complete remission with androgen-deprivation therapy in a recurrent androgen receptor-expressing adenocarcinoma of the parotid gland. Ann Oncol 2003;14(8):1327-8.
8. Kuroda H, Sakurai T, Yamada M, *et al.* [Effective treatment by both anti-androgen therapy and chemotherapy for a patient with advanced salivary duct carcinoma]. Gan To Kagaku Ryoho 2011;38(4):627-30.
9. Rajoria S, Suriano R, George A, *et al.* Estrogen induced metastatic modulators MMP-2 and MMP-9 are targets of 3,3'-diindolylmethane in thyroid cancer. PLoS One 2011;6(1):e15879.
10. Rahbari R, Zhang L, Kebebew E. Thyroid cancer gender disparity. Future Oncol 2010;6(11):1771-9.
11. Negri E, Ron E, Franceschi S, *et al.* A pooled analysis of case-control studies of thyroid cancer. I. Methods. Cancer Causes Control 1999;10(2):131-42.
12. Blechet C, Lecomte P, De Calan L, *et al.* Expression of sex steroid hormone receptors in C cell hyperplasia and medullary thyroid carcinoma. Virchows Arch 2007;450(4):433-9.
13. Banu KS, Govindarajulu P, Aruldhas MM. Testosterone and estradiol have specific differential modulatory effect on the proliferation of human thyroid papillary and follicular carcinoma cell lines independent of TSH action. Endocr Pathol 2001;12(3):315-27.
14. Wiseman SM, Melck A, Masoudi H, *et al.* Molecular phenotyping of thyroid tumors identifies a marker panel for differentiated thyroid cancer diagnosis. Ann Surg Oncol 2008;15(10):2811-26.
15. Prinz RA, Sandberg L, Chaudhuri PK. Androgen receptors in human thyroid tissue. Surgery. 1984 Dec;96(6):996-1000.
16. Lemon HM, Ravin IS, Ross JF, *et al.* Testosterone therapy of metastatic adenocarcinoma of the thyroid, with remission; case report. Cancer 1951;4(6):1176-92.
17. Ishibashi H, Suzuki T, Suzuki S, *et al.* Sex steroid hormone receptors in human thymoma. J Clin Endocrinol Metab 2003;88(5):2309-17.
18. Mimae T, Tsuta K, Takahashi F, *et al.* Steroid receptor expression in thymomas and thymic carcinomas. Cancer 2011;117(19):4396-405.

19. Khoury T, Chandrasekhar R, Wilding G, *et al.* Tumour eosinophilia combined with an immunohistochemistry panel is useful in the differentiation of type B3 thymoma from thymic carcinoma. Int J Exp Pathol 2011;92(2):87-96.
20. Chapman MS, Askew DJ, Kuscuoglu U, *et al.* Transcriptional control of steroid-regulated apoptosis in murine thymoma cells. Mol Endocrinol 1996;10(8):967-78.
21. Hagen R, Schweikert HU, Schwab B, *et al.* [Hormone dependence of juvenile nasopharyngeal fibroma]. Laryngorhinootologie 1991;70(6):279-83.
22. Brentani MM, Butugan O, Oshima CT, *et al.* Multiple steroid receptors in nasopharyngeal angiofibromas. Laryngoscope 1989;99(4):398-401.
23. Hwang HC, Mills SE, Patterson K, *et al.* Expression of androgen receptors in nasopharyngeal angiofibroma: an immunohistochemical study of 24 cases. Mod Pathol 1998;11(11):1122-6.
24. Montag AG, Tretiakova M, Richardson M. Steroid hormone receptor expression in nasopharyngeal angiofibromas. Consistent expression of estrogen receptor beta. Am J Clin Pathol 2006;125(6):832-7.
25. Gates GA, Rice DH, Koopmann CF, Jr., *et al.* Flutamide-induced regression of angiofibroma. Laryngoscope 1992;102(6):641-4.
26. Bianchini C, Pastore A, Pelucchi S, *et al.* Sex hormone receptor levels in laryngeal carcinoma: a comparison between protein and RNA evaluations. Eur Arch Otorhinolaryngol 2008;265(9):1089-94.
27. Virolainen E, Vanharanta R, Carey TE. Steroid hormone receptors in human squamous carcinoma cell lines. Int J Cancer 1984;33(1):19-25.
28. Mattox DE, Von Hoff DD, McGuire WL. Androgen receptors and antiandrogen therapy for laryngeal carcinoma. Arch Otolaryngol 1984;110(11):721-4.
29. Kokoschka EM, Spona J, Knobler R. Sex steroid hormone receptor analysis in malignant melanoma. Br J Dermatol 1982;107 Suppl 23:54-9.
30. Gershtein ES, Tailakov BB, Smirnova KD, *et al.* [Cytoplasmic receptors of steroid hormones and clinico-morphological characteristics of skin melanoma]. Vopr Onkol 1991;37(4):441-6.
31. Morvillo V, Luthy IA, Bravo AI, *et al.* Atypical androgen receptor in the human melanoma cell line IIB-MEL-J. Pigment Cell Res 1995;8(3):135-41.
32. Morvillo V, Luthy IA, Bravo AI, *et al.* Androgen receptors in human melanoma cell lines IIB-MEL-LES and IIB-MEL-IAN and in human melanoma metastases. Melanoma Res 2002;12(6):529-38.
33. Nardone PA, Slotman GJ, Vezeridis MP. Ketoconazole: a thromboxane synthetase and 5-lipoxygenase inhibitor with antimetastatic activity in B16-F10 melanoma. J Surg Res 1988;44(4):425-9.
34. Katona TM, Perkins SM, Billings SD. Does the panel of cytokeratin 20 and androgen receptor antibodies differentiate desmoplastic trichoepithelioma from morpheaform/infiltrative basal cell carcinoma? J Cutan Pathol 2008;35(2):174-9.
35. Izikson L, Bhan A, Zembowicz A. Androgen receptor expression helps to differentiate basal cell carcinoma from benign trichoblastic tumors. Am J Dermatopathol 2005;27(2):91-5.
36. Dohi O, Hatori M, Suzuki T, *et al.* Sex steroid receptors expression and hormone-induced cell proliferation in human osteosarcoma. Cancer Sci 2008;99(3):518-23.
37. Liu J, Chen X, Li S, *et al.* [Expression and significance of AR in osteosarcoma]. Zhonghua Wai Ke Za Zhi 2000;38(5):344-5, 23.
38. Lin KL, Huang CC, Cheng JS, *et al.* Ketoconazole-induced JNK phosphorylation and subsequent cell death via apoptosis in human osteosarcoma cells. Toxicol In Vitro 2009;23(7):1268-76.
39. Brodsky SV, Gimenez C, Ghosh C, *et al.* Estrogen and progesterone receptors expression in gastrointestinal stromal tumors and intramural gastrointestinal leiomyomas. Int J Gastrointest Cancer 2006;37(4):129-32.
40. Lam MM, Corless CL, Goldblum JR, *et al.* Extragastrointestinal stromal tumors presenting as vulvovaginal/rectovaginal septal masses: a diagnostic pitfall. Int J Gynecol Pathol 2006;25(3):288-92.
41. Walker MJ, Chaudhuri PK, Beattie CW, *et al.* Steroid receptors in malignant skeletal tumors. Cancer 1980;45(12):3004-7.

42. Ziegler JL, Katongole-Mbidde E, Wabinga H, *et al.* Absence of sex-hormone receptors in Kaposi's sarcoma. Lancet 1995;345(8954):925.
43. Pais V, Leav I, Lau KM, *et al.* Estrogen receptor-beta expression in human testicular germ cell tumors. Clin Cancer Res 2003;9(12):4475-82.
44. Rajpert-De Meyts E, Skakkebaek NE. Immunohistochemical identification of androgen receptors in germ cell neoplasia. J Endocrinol 1992;135(2):R1-4.
45. Langner C, Ratschek M, Rehak P, *et al.* Steroid hormone receptor expression in renal cell carcinoma: an immunohistochemical analysis of 182 tumors. J Urol 2004;171(2 Pt 1):611-4.
46. Brown DF, Dababo MA, Hladik CL, *et al.* Hormone receptor immunoreactivity in hemangioblastomas and clear cell renal cell carcinomas. Mod Pathol 1998;11(1):55-9.
47. Klotzl G, Otto U, Becker H, *et al.* Determination of androgen, progestin and estrogen receptors with two different assays in renal cell carcinoma. Urol Int 1987;42(2):100-4.
48. Veytsman I, Nieman L, Fojo T. Management of endocrine manifestations and the use of mitotane as a chemotherapeutic agent for adrenocortical carcinoma. J Clin Oncol 2009;27(27):4619-29.
49. Barzon L, Masi G, Pacenti M, *et al.* Expression of aromatase and estrogen receptors in human adrenocortical tumors. Virchows Arch 2008;452(2):181-91.
50. Rossi R, Zatelli MC, Valentini A, *et al.* Evidence for androgen receptor gene expression and growth inhibitory effect of dihydrotestosterone on human adrenocortical cells. J Endocrinol 1998;159(3):373-80.
51. Harinarayan CV, Gupta S, Ammini AC, *et al.* Clinical utility of ketoconazole in cases of adrenocortical carcinoma. Indian J Cancer 1991;28(4):196-201.
52. Verhelst JA, Druwe P, van Erps P, *et al.* Use of ketoconazole in the treatment of a virilizing adrenocortical carcinoma. Acta Endocrinol (Copenh) 1989;121(2):229-34.
53. Mir C, Shariat SF, van der Kwast TH, *et al.* Loss of androgen receptor expression is not associated with pathological stage, grade, gender or outcome in bladder cancer: a large multi-institutional study. BJU Int 2011;108(1):24-30.
54. Miyamoto H, Yao JL, Chaux A, *et al.* Expression of androgen and oestrogen receptors and its prognostic significance in urothelial neoplasm of the urinary bladder. BJU Int 2012;109(11):1716-26.
55. Tuygun C, Kankaya D, Imamoglu A, *et al.* Sex-specific hormone receptors in urothelial carcinomas of the human urinary bladder: a comparative analysis of clinicopathological features and survival outcomes according to receptor expression. Urol Oncol 2011;29(1):43-51.
56. Miyamoto H, Yang Z, Chen YT, *et al.* Promotion of bladder cancer development and progression by androgen receptor signals. J Natl Cancer Inst 2007;99(7):558-68.
57. Hameed DA, el-Metwally TH. The effectiveness of retinoic acid treatment in bladder cancer: impact on recurrence, survival and TGFalpha and VEGF as end-point biomarkers. Cancer Biol Ther 2008;7(1):92-100.
58. Leav I, Lau KM, Adams JY, *et al.* Comparative studies of the estrogen receptors beta and alpha and the androgen receptor in normal human prostate glands, dysplasia, and in primary and metastatic carcinoma. Am J Pathol 2001;159(1):79-92.
59. Gustafsson JA, Ekman P, Snochowski M, *et al.* Correlation between clinical response to hormone therapy and steroid receptor content in prostatic cancer. Cancer Res 1978;38(11 Pt 2):4345-8.
60. Ekman P, Gustafsson JA, Hogberg B, *et al.* Prediction of tumor response to endocrine therapy in prostatic carcinoma based on steroid receptor assay. Scand J Urol Nephrol Suppl 1980;55:83-9.
61. Bassalyk LS, Petrova AS, Ermilova VD, *et al.* [Relationship between androgen and estrogen receptors and clinico-morphological characteristics of prostatic cancer]. Arkh Patol 1983;45(2):23-8.
62. Kaiser U, Hofmann J, Schilli M, *et al.* Steroid-hormone receptors in cell lines and tumor biopsies of human lung cancer. Int J Cancer 1996;67(3):357-64.
63. Rades D, Setter C, Dahl O, *et al.* The prognostic impact of tumor cell expression of estrogen receptor-alpha, progesterone receptor, and androgen receptor in patients irradiated for nonsmall cell lung cancer. Cancer 2012;118(1):157-63.

64. Maasberg M, Rotsch M, Jaques G, *et al.* Androgen receptors, androgen-dependent proliferation, and 5 alpha-reductase activity of small-cell lung cancer cell lines. Int J Cancer 1989;43(4):685-91.
65. Wu CW, Chi CW, Chang TJ, *et al.* Sex hormone receptors in gastric cancer. Cancer 1990;65(6):1396-400.
66. Kominea A, Konstantinopoulos PA, Kapranos N, *et al.* Androgen receptor (AR) expression is an independent unfavorable prognostic factor in gastric cancer. J Cancer Res Clin Oncol 2004;130(5):253-8.
67. Nakamura Y, Shimada N, Suzuki T, *et al.* In situ androgen production in human gastric carcinoma--androgen synthesizing and metabolizing enzymes. Anticancer Res 2006;26(3A):1935-9.
68. Yamashita Y, Hirai T, Mukaida H, *et al.* Detection of androgen receptors in human esophageal cancer. Jpn J Surg 1989;19(2):195-202.
69. Tihan T, Harmon JW, Wan X, *et al.* Evidence of androgen receptor expression in squamous and adenocarcinoma of the esophagus. Anticancer Res 2001;21(4B):3107-14.
70. Cooper SC, Trudgill NJ. Subjects with prostate cancer are less likely to develop esophageal cancer: analysis of SEER 9 registries database. Cancer Causes Control 2012;23(6):819-25.
71. Corbishley TP, Iqbal MJ, Wilkinson ML, *et al.* Androgen receptor in human normal and malignant pancreatic tissue and cell lines. Cancer 1986;57(10):1992-5.
72. Targarona EM, Pons MD, Gonzalez G, *et al.* Is exocrine pancreatic cancer a hormone-dependent tumor? A study of the existence of sex hormone receptors in normal and neoplastic pancreas. Hepatogastroenterology 1991;38(2):165-9.
73. Sharma JJ, Razvillas B, Stephens CD, *et al.* Phase II study of flutamide as second line chemotherapy in patients with advanced pancreatic cancer. Invest New Drugs 1997;15(4):361-4.
74. Greenway BA. Effect of flutamide on survival in patients with pancreatic cancer: results of a prospective, randomised, double blind, placebo controlled trial. BMJ 1998;316(7149):1935-8.
75. Andren-Sandberg A, Hoem D, Backman PL. Other risk factors for pancreatic cancer: hormonal aspects. Ann Oncol 1999;10 Suppl 4:131-5.
76. Konduri S, Schwarz MA, Cafasso D, *et al.* Androgen receptor blockade in experimental combination therapy of pancreatic cancer. J Surg Res 2007;142(2):378-86.
77. Wibowo E, Schellhammer P, Wassersug RJ. Role of estrogen in normal male function: clinical implications for patients with prostate cancer on androgen deprivation therapy. J Urol 2011;185(1):17-23.
78. Sun M, Estrov Z, Ji Y, *et al.* Curcumin (diferuloylmethane) alters the expression profiles of microRNAs in human pancreatic cancer cells. Mol Cancer Ther 2008;7(3):464-73.
79. Tutton PJ, Barkla DH. The influence of androgens, anti-androgens, and castration on cell proliferation in the jejunal and colonic crypt epithelia, and in dimethylhydrazine-induced adenocarcinoma of rat colon. Virchows Arch B Cell Pathol Incl Mol Pathol 1982;38(3):351-5.
80. Stebbings WS, Farthing MJ, Vinson GP, *et al.* Androgen receptors in rectal and colonic cancer. Dis Colon Rectum 1986;29(2):95-8.
81. Meggouh F, Lointier P, Pezet D, *et al.* Status of sex steroid hormone receptors in large bowel cancer. Cancer 1991;67(7):1964-70.
82. Berta L, Fronticelli Baldelli C, Fazzari A, *et al.* Sex steroid receptors, secondary bile acids and colorectal cancer. A possible mechanism of interaction. Panminerva Med 2003;45(4):261-6.
83. Castagnetta L, Traina A, Campisi I, *et al.* Androgen receptor status in nontumoral and malignant human colorectal tissues. Ann N Y Acad Sci 2002;963:322-5.
84. Forgue-Lafitte ME, Coudray AM, Fagot D, *et al.* Effects of ketoconazole on the proliferation and cell cycle of human cancer cell lines. Cancer Res 1992;52(24):6827-31.
85. Ishizuka M, Hatori M, Dohi O, *et al.* Expression profiles of sex steroid receptors in desmoid tumors. Tohoku J Exp Med 2006;210(3):189-98.
86. van Dam JH, Schouten WR, Kok TC. [Treatment and course of desmoid tumors in 5 patients with familial adenomatous polyposis]. Ned Tijdschr Geneeskd 1993;137(14):716-20.
87. Bauernhofer T, Stoger H, Schmid M, *et al.* Sequential treatment of recurrent mesenteric desmoid tumor. Cancer 1996;77(6):1061-5.

88. Merritt RL, Foran CM. Influence of persistent contaminants and steroid hormones on glioblastoma cell growth. J Toxicol Environ Health A 2007;70(1):19-27.
89. Chung YG, Kim HK, Lee HK, *et al.* Expression of androgen receptors in astrocytoma. J Korean Med Sci 1996;11(6):517-21.
90. Lee LS, Chi CW, Chang TJ, *et al.* Steroid hormone receptors in meningiomas of Chinese patients. Neurosurgery 1989;25(4):541-5.
91. Noel JC, Bucella D, Fayt I, *et al.* Androgen receptor expression in cervical intraepithelial neoplasia and invasive squamous cell carcinoma of the cervix. Int J Gynecol Pathol 2008;27(3):437-41.
92. Yang HP, Gonzalez Bosquet J, Li Q, *et al.* Common genetic variation in the sex hormone metabolic pathway and endometrial cancer risk: pathway-based evaluation of candidate genes. Carcinogenesis 2010;31(5):827-33.
93. Ito K, Suzuki T, Akahira J, *et al.* Expression of androgen receptor and 5alpha-reductases in the human normal endometrium and its disorders. Int J Cancer 2002;99(5):652-7.
94. Brys M, Semczuk A, Baranowski W, *et al.* Androgen receptor (AR) expression in normal and cancerous human endometrial tissues detected by RT-PCR and immunohistochemistry. Anticancer Res 2002;22(2A):1025-31.
95. Koivisto-Korander R, Butzow R, Koivisto AM, *et al.* Immunohistochemical studies on uterine carcinosarcoma, leiomyosarcoma, and endometrial stromal sarcoma: expression and prognostic importance of ten different markers. Tumour Biol 2011;32(3):451-9.
96. Moinfar F, Regitnig P, Tabrizi AD, *et al.* Expression of androgen receptors in benign and malignant endometrial stromal neoplasms. Virchows Arch 2004;444(5):410-4.
97. Leitao MM, Soslow RA, Nonaka D, *et al.* Tissue microarray immunohistochemical expression of estrogen, progesterone, and androgen receptors in uterine leiomyomata and leiomyosarcoma. Cancer 2004;101(6):1455-62.
98. al-Timimi A, Buckley CH, Fox H. An immunohistochemical study of the incidence and significance of sex steroid hormone binding sites in normal and neoplastic human ovarian tissue. Int J Gynecol Pathol 1985;4(1):24-41.
99. Elattar A, Warburton KG, Mukhopadhyay A, *et al.* Androgen receptor expression is a biological marker for androgen sensitivity in high grade serous epithelial ovarian cancer. Gynecol Oncol 2012;124(1):142-7.
100. Jones MW, Harri R, Dabbs DJ, *et al.* Immunohistochemical profile of steroid cell tumor of the ovary: a study of 14 cases and a review of the literature. Int J Gynecol Pathol 2010;29(4):315-20.
101. Nodin B, Zendehrokh N, Brandstedt J, *et al.* Increased androgen receptor expression in serous carcinoma of the ovary is associated with an improved survival. J Ovarian Res 2010;3:14.
102. Dizon DS, Tejada-Berges T, Koelliker S, *et al.* Ovarian cancer associated with testosterone supplementation in a female-to-male transsexual patient. Gynecol Obstet Invest 2006;62(4):226-8.
103. Levine D, Park K, Juretzka M, *et al.* A phase II evaluation of goserelin and bicalutamide in patients with ovarian cancer in second or higher complete clinical disease remission. Cancer 2007;110(11):2448-56.
104. Wang PH, Chang C. Androgens and ovarian cancers. Eur J Gynaecol Oncol 2004;25(2):157-63.
105. van Doorn HC, Burger CW, van der Valk P, *et al.* Oestrogen, progesterone, and androgen receptors in ovarian neoplasia: correlation between immunohistochemical and biochemical receptor analyses. J Clin Pathol 2000;53(3):201-5.
106. Tumolo S, Rao BR, van der Burg ME, *et al.* Phase II trial of flutamide in advanced ovarian cancer: an EORTC Gynaecological Cancer Cooperative Group study. Eur J Cancer 1994;30A(7):911-4.
107. Klotz RK, Muller-Holzner E, Fessler S, *et al.* Leydig-cell-tumor of the ovary that responded to GnRH-analogue administration - case report and review of the literature. Exp Clin Endocrinol Diabetes 2010;118(5):291-7.
108. Papadatos-Pastos D, Dedes KJ, de Bono JS, *et al.* Revisiting the role of antiandrogen strategies in ovarian cancer. Oncologist 2011;16(10):1413-21.

109. Peters KM, Edwards SL, Nair SS, *et al.* Androgen receptor expression predicts breast cancer survival: the role of genetic and epigenetic events. BMC Cancer 2012;12:132.
110. Lin Fde M, Pincerato KM, Bacchi CE, *et al.* Coordinated expression of oestrogen and androgen receptors in HER2-positive breast carcinomas: impact on proliferative activity. J Clin Pathol 2012;65(1):64-8.
111. Hu R, Dawood S, Holmes MD, *et al.* Androgen receptor expression and breast cancer survival in postmenopausal women. Clin Cancer Res 2011;17(7):1867-74.
112. Pristauz G, Petru E, Stacher E, *et al.* Androgen receptor expression in breast cancer patients tested for BRCA1 and BRCA2 mutations. Histopathology 2010;57(6):877-84.
113. Qi JP, Yang YL, Zhu H, *et al.* Expression of the androgen receptor and its correlation with molecular subtypes in 980 chinese breast cancer patients. Breast Cancer (Auckl) 2012;6:1-8.
114. Cimino-Mathews A, Hicks JL, Illei PB, *et al.* Androgen receptor expression is usually maintained in initial surgically resected breast cancer metastases but is often lost in end-stage metastases found at autopsy. Hum Pathol 2012;43(7):1003-11.
115. Collins LC, Cole KS, Marotti JD, *et al.* Androgen receptor expression in breast cancer in relation to molecular phenotype: results from the Nurses' Health Study. Mod Pathol 2011;24(7):924-31.
116. Lehmann BD, Bauer JA, Chen X, *et al.* Identification of human triple-negative breast cancer subtypes and preclinical models for selection of targeted therapies. J Clin Invest 2011;121(7):2750-67.
117. Doane AS, Danso M, Lal P, *et al.* An estrogen receptor-negative breast cancer subset characterized by a hormonally regulated transcriptional program and response to androgen. Oncogene 2006;25(28):3994-4008.
118. De Amicis F, Thirugnansampanthan J, Cui Y, *et al.* Androgen receptor overexpression induces tamoxifen resistance in human breast cancer cells. Breast Cancer Res Treat 2010;121(1):1-11.
119. Gucalp A, Tolaney SM, Isakoff SJ, *et al.* Targeting the androgen receptor (AR) in women with AR+ ER-/PR- metastatic breast cancer (MBC). J Clin Oncol 2012;30(Suppl_2):1006.
